# Plasma MMP1 and MMP8 expression in breast cancer: Protective role of MMP8 against lymph node metastasis

**DOI:** 10.1186/1471-2407-8-77

**Published:** 2008-03-20

**Authors:** Julie Decock, Wouter Hendrickx, Ulla Vanleeuw, Vanya Van Belle, Sabine Van Huffel, Marie-Rose Christiaens, Shu Ye, Robert Paridaens

**Affiliations:** 1 Laboratory for Experimental Oncology (LEO), K.U.Leuven, Campus University Hospital Gasthuisberg, O&N1 bus 815, Herestraat 49, 3000 Leuven, Belgium; 2 Multidisciplinary Breast Centre (MBC), University Hospitals Leuven, Herestraat 49, 3000 Leuven, Belgium; 3 Department of Electrical Engineering (ESAT), Division SCD, K.U.Leuven, Kasteelpark Arenberg 10 bus 2446, 3001 Leuven, Belgium; 4 Clinical Pharmacology, William Harvey Research Institute, John Vane Science Building, Charthouse Square, London EC1M 6BQ, UK

## Abstract

**Background:**

Elevated levels of matrix metalloproteinases have been found to associate with poor prognosis in various carcinomas. This study aimed at evaluating plasma levels of MMP1, MMP8 and MMP13 as diagnostic and prognostic markers of breast cancer.

**Methods:**

A total of 208 breast cancer patients, of which 21 with inflammatory breast cancer, and 42 healthy controls were included. Plasma MMP1, MMP8 and MMP13 levels were measured using ELISA and correlated with clinicopathological characteristics.

**Results:**

Median plasma MMP1 levels were higher in controls than in breast cancer patients (3.45 vs. 2.01 ng/ml), while no difference was found for MMP8 (10.74 vs. 10.49 ng/ml). ROC analysis for MMP1 revealed an AUC of 0.67, sensitivity of 80% and specificity of 24% at a cut-off value of 4.24 ng/ml. Plasma MMP13 expression could not be detected. No correlation was found between MMP1 and MMP8 levels. We found a trend of lower MMP1 levels with increasing tumour size (p = 0.07); and higher MMP8 levels with premenopausal status (p = 0.06) and NPI (p = 0.04). The median plasma MMP1 (p = 0.02) and MMP8 (p = 0.007) levels in the non-inflammatory breast cancer patients were almost twice as high as those found in the inflammatory breast cancer patients. Intriguingly, plasma MMP8 levels were positively associated with lymph node involvement but showed a negative correlation with the risk of distant metastasis. Both controls and lymph node negative patients (pN0) had lower MMP8 levels than patients with moderate lymph node involvement (pN1, pN2) (p = 0.001); and showed a trend for higher MMP8 levels compared to patients with extensive lymph node involvement (pN3) and a strong predisposition to distant metastasis (p = 0.11). Based on the hypothesis that blood and tissue protein levels are in reverse association, these results suggest that MMP8 in the tumour may have a protective effect against lymph node metastasis.

**Conclusion:**

In summary, we observed differences in MMP1 and MMP8 plasma levels between healthy controls and breast cancer patients as well as between breast cancer patients. Interestingly, our results suggest that MMP8 may affect the metastatic behaviour of breast cancer cells through protection against lymph node metastasis, underlining the importance of anti-target identification in drug development.

## Background

Invasive breast cancer is one of the most common forms of cancer in Europe. In women, breast cancer accounts for 29% of all cancers and is the major cause of cancer deaths [1]. Currently, prognosis and choice of treatment is determined based on histologic tumour type, grade, tumour size, lymph node involvement, steroid hormone receptor expression and Her2 status. However, assessment of these clinical and pathological features does not enable us to fully capture the heterogeneous clinical course of breast cancer [2]. Therefore, there is a continuous search for new biomarkers of invasion and metastases.

Matrix metalloproteinases (MMPs) are a family of zinc dependent endopeptidases that can degrade virtually all extracellular matrix components [3]. Five MMP subgroups are defined according to their substrate specificity and domain structure: collagenases, gelatinases, stromelyins, matrilysins and membrane-type MMPs. Both in physiological and pathological conditions their expression is rapidly induced when active tissue remodelling is needed. It is well recognized that MMPs are key mediators of tumour invasion and metastasis, being involved in cell proliferation, survival, angiogenesis, and cell migration [4]. Aberrant MMP expression has been observed to be associated with prognosis in several types of carcinomas, including breast cancer. Because tissue MMPs may enter into the blood stream and increase circulating levels, it is believed that MMP levels in the blood may serve as biological markers for disease onset or progression, and allow monitoring of the disease. Several studies have evaluated the diagnostic and prognostic value of circulating MMP2 and MMP9 in breast cancer because of their important role in tumour invasion and metastasis. Elevated levels of both MMP2 and MMP9 have been observed in blood from breast cancer patients and were repeatedly found to be associated with advanced stage, lymph node metastasis and poor prognosis [5-15].

In this study, we evaluated the usefulness of circulating MMP1, MMP8 and MMP13 levels as diagnostic and prognostic markers in breast cancer. These MMPs form the collagenase subgroup of MMPs, capable of cleaving native fibrillar collagens (types I, II, III, and V) as well as various non-collagenous molecules [16]. Upregulation of all three MMPs has been demonstrated in several tumours [16-18]. As yet, however, little is known about their diagnostic and prognostic value in tumours and blood from breast cancer patients. We used plasma samples to determine circulating collagenase levels since an increasing number of reports indicates that it is the most reliable source for MMP quantification in blood. For instance, serum MMP2 and MMP9 levels are found to be 'artificially' higher than plasma levels due to increased release from platelets and leukocytes during coagulation [19]. Since MMP8 is mainly synthesized and stored in neutrophils, serum MMP8 levels may also be affected by blood sampling and handling. In order to avoid unreliable results, we thus determined the MMP1, MMP8 and MMP13 expression in plasma. This study aimed to evaluate 1) the diagnostic value of circulating MMP1, MMP8 and MMP13 in breast cancer patients, 2) the prognostic value of plasma MMP1, MMP8 and MMP13 in breast cancer by correlating their levels with standard prognostic factors, 3) the diagnostic value of circulating MMP1, MMP8 and MMP13 in inflammatory breast cancer which is characterized by a particularly aggressive behaviour and poor prognosis [20].

## Methods

### Subjects

A group of 208 patients with invasive breast cancer, newly diagnosed at the Multidisciplinary Breast Centre of the University hospital Leuven between 2003 and 2007, were prospectively recruited to this study. The study design was approved by the Medical Ethical Committee of the University Hospital Leuven. Patient characteristics were extracted from clinical files, tumour characteristics and lymph node status were retrieved from the pathology reports, and all data were eventually collected in one central database. In order for patients to be included in the study the presence and appropriate storage of plasma samples at the time of diagnosis was required. Patients with a history of cancer, bilateral cancer or Paget's disease of the nipple were excluded from the study. Twenty one patients of the study group presented with inflammatory breast cancer at diagnosis and all received neo-adjuvant treatment according to the standard treatment guidelines of our institute at that time. Therefore, their data on tumour size, histologic grade, steroid hormone receptor expression, Human Epidermal growth factor Receptor 2 (Her2) status, Nottingham Prognostic Index (NPI) and lymph node involvement were not included in the statistical analyses as they may be affected by treatment. Non-inflammatory breast cancer patients had not received any treatment at the time of diagnosis. The study also included 42 age-matched healthy controls, attending the Blood Transfusion Centre in Leuven.

### Plasma samples

Blood samples were collected into EDTA coated plasma collection tubes. Plasma was obtained by centrifugation at 1300 g for 10 min and stored at -80°C until use. All personal identifiers of healthy controls and patients were removed prior to transfer of samples to the biobank of the Multidisciplinary Breast Centre of Leuven.

### Histology of primary tumours

Tumour tissue was snap-frozen in liquid nitrogen within 1 hour after surgery. Typing of primary tumours was performed according to the WHO-classification while for grading the Ellis and Elston system was used. Lymph nodes were examined with haematoxylin eosin using 3 sections per node and sentinel lymph nodes classified as negative were additionally stained with epithelial markers. Immunohistochemical staining for the oestrogen receptor (ER), progesterone receptor (PR) and Her2 was performed on formalin-fixed paraffin-embedded 4 μm thick tissue sections. Staining of the steroid hormone receptors was semi-quantitatively evaluated using the Allred score and samples with a score of 3 or more were respectively defined ER or PR positive. The DAKO-HercepTest scoring system was used to evaluate Her2 staining, after which cases with an intermediary staining (2+) were further analyzed by dual-colour Fluorescence in situ Hybridization in order to distinguish true Her2 gene amplification from polysomy 17.

### Sandwich enzyme-linked immunoassays (sELISAs)

Plasma MMP1, MMP8 and MMP13 levels were measured using specific sELISAs, detecting both the pro- and active forms (GE Healthcare Europe GmbH, Munich, Germany for MMP1 and MMP13; RnD Systems Europe Ltd., Abingdon, United Kingdom for MMP8). Plasma samples were allowed to thaw at room temperature just before use. Samples were diluted 20-fold for MMP8 analysis, while for MMP1 and MMP13 measurements undiluted plasma was used. Not all plasma samples were examined for MMP1, MMP8 as well as MMP13 due to sample volume deficiency or technical problems with some samples. Measurements were done in duplicate and results were expressed as median (interquartile range).

### Statistical analysis

Assumption of normality was verified using the normal probability plot, Shapiro-Wilk's W test and the Levene's test for homogeneity of variances. None of the plasma collagenase levels were found to be normally distributed. Measurements were done in duplicate and results were expressed as median, 1^st ^and 3^rd ^quartile or interquartile range. The Mann-Whitney U test and Kruskal-Wallis test were used to assess differences in plasma MMP1 and MMP8 levels between healthy controls and patients as well as between different patient groups. Odds ratios (OR) were also calculated for the latter. Correlations were evaluated using Spearman rank correlation coefficients. The diagnostic values of plasma collagenase levels for breast cancer were evaluated by receiver-operating characteristics (ROC) analysis. All statistical analyses were carried out using the software package SAS 9.1.3 service pack 4, the level of significance being set at α = 0,05.

## Results

### Subject characteristics

In total 208 patients were included in the study. Data for MMP1 levels were unavailable for 6 patients and MMP8 data were unavailable for 10 patients, due to insufficient sample volume or technical problems. The mean age at diagnosis was 57 years (min-max; 28–86). Seventy four (36%) patients were premenopausal, 123 (59%) postmenopausal and for 11 (5%) patients menopausal status was unknown.

Twenty one (10%) patients had inflammatory breast cancer and were not included for association analyses of tumour size, histologic grade, lymph node involvement, steroid hormone receptor status, Her2 expression and NPI value. Most non-inflammatory carcinomas were classified pT1 (43%; 81/187) or pT2 (42%; 78/187), and only a minority was categorized as pT3 (15%; 28/187). Half (53%; 99/187) of all tumours were poorly differentiated, 38% (71/187) moderately and 9% (17/187) well. A total of 48% (89/187) of all patients were free of lymph node metastasis, 36% (68/187) were affected to the extent of stage pN1, 10% (19/187) to pN2 and 6% (11/187) to pN3. Of all 187 non-inflammatory breast tumours, 12% (22/187) were ER negative, 88% (165/187) ER positive, 18% (33/187) PR negative and 82% (153/187) PR positive. The vast majority (87%; 163/187) of all tumours showed no overexpression of Her2.

### Plasma collagenase levels in patients and healthy controls

**Figure 1:**
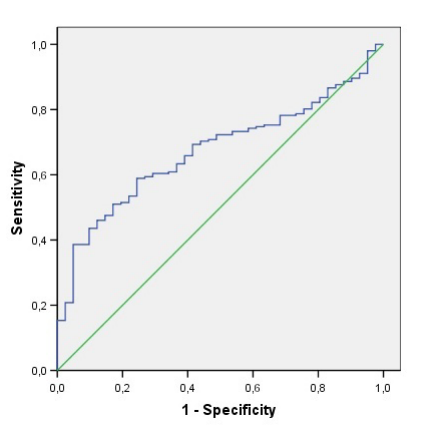
Receiver-operator curves for plasma MMP1 levels, with AUC of 0.67.

Median plasma MMP1 levels were significantly higher (p = 0.0005) in healthy controls (3.45 ng/ml, interquartile range IQR 2.42–4.17 ng/ml) than in breast cancer patients (2.01 ng/ml, IQR 0.79 – 3.71 ng/ml). A ROC curve was generated for further analysis of the diagnostic value of MMP1, as presented in Figure 1. The area under the curve (AUC) was 0.67 (95% confidence interval 0.60 – 0.75), with a sensitivity of 80% and specificity of 24% at a cut-off value of 4.24 ng/ml. The highest diagnostic accuracy, defined by a minimal number of false negative and false positives, was found at a cut-off value of 2.40 ng/ml with a sensitivity of 56% and specificity of 76%. No difference in MMP8 levels was found between controls (10.74 ng/ml, IQR 7.47 – 15.3 ng/ml) and patients (10.49 ng/ml, IQR 4.22 – 18.93 ng/ml), the lack of diagnostic value of plasma MMP8 was confirmed by ROC analysis with a AUC of 0.53 (data not shown).

Plasma MMP1 and MMP8 levels did not correlate among controls (rs = 0.10, p = 0.54, data not shown) nor among breast cancer patients (rs = 0.009, p = 0.90, data not shown).

Plasma MMP-13 levels could not be determined because they did not reach the detection sensitivity of the assay in both breast cancer patients and controls.

### Plasma MMP1 and MMP8 expression in relation to clinicopathological parameters

**Figure 2:**
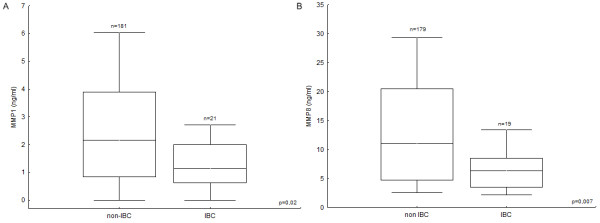
Differences in plasma levels of **A) **MMP1 and **B) **MMP8 between patients with and without inflammatory breast cancer (box-whisker diagrams with median, 1^st ^quartile, 3^rd ^quartile and non-outlier range).

The associations of plasma MMP1 and MMP8 levels with various clinical and pathological variables are presented in Table 1. We observed a trend of lower plasma MMP1 levels with increasing tumour size (Odds ratio OR 1.11, p = 0.07) and found significantly lower MMP1 expression in patients with inflammatory breast cancer (OR 0.74, p = 0.02, Figure 2a).

**Table 1:** Plasma MMP1 and MMP8 levels in relation to standard prognostic markers.

Characteristics	MMP1 (ng/ml)		OR (95% CI)	p*	MMP8 (ng/ml)		OR (95% CI)	p*
	Median (IQR)	n			Median (IQR)	n		

*all patients*	2.0 (0.8–3.7)	202			10.5 (4.4–19.1)	198		
								
*menopausal status*								
premenopausal	2.0 (0.5–4.0)	70	1.02 (0.91–1.13)	0.67	14.8 (5.4–20.8)	70	0.98 (0.95–1.00)	0.06
postmenopausal	2.0 (1.0–3.5)	122			10.0 (4.2–18.8)	117		
unknown	2.2 (1.3–3.0)	10			6.0 (3.6–13.4)	11		
								
*breast cancer*								
inflammatory	1.1 (0.6–2.0)	21	0.74 (0.56–0.98)	0.02	6.3 (3.5–8.5)	19	0.90 (0.83–0.97)	0.007
non-inflammatory	2.2 (0.8–3.9)	181			11.0 (4.7–20.5)	179		
								
*non-inflammatory breast cancer patients*								
								
*Primary tumour size*								
pT1	2.4 (1.1–4.2)	79	1.11 (1.00–1.23)	0.07	10.2 (3.8–20.5)	76	0.99 (0.97–1.01)	0.14
pT2	2.2 (0.8–3.7)	76			11.0 (4.6–18.8)	77		
pT3	1.3 (0.6–2.2)	26			16.4 (6.4–24.0)	26		
								
*axillary lymph node status*								
pN0	2.3 (1.1–4.1)	86	1.04 (0.94–1.15)	0.18	7.5 (3.5–19.1)	83	0.99 (0.97–1.01)	0.003
pN1	1.9 (0.8–3.6)	67			13.4 (8.0–22.5)	66		
pN2	0.9 (0.1–3.4)	19			18.0 (12.5–22.0)	19		
pN3	2.7 (1.9–4.6)	9			5.7 (2.9–8.9)	11		
								
*histologic grade*								
well (G1)	3.4 (1.0–4.6)	17	0.99 (0.90–1.10)	0.14	4.9 (3.3–20.9)	16	1.00 (0.98–1.03)	0.37
moderatly (G2)	1.6 (0.6–3.0)	70			15.2 (4.8–21.6)	68		
poorly (G3)	2.2 (1.1–4.0)	94			11.0 (5.4–18.9)	95		
								
*oestrogen receptor*								
Negative	1.6 (0.2–3.7)	21	1.13 (0.92–1.39)	0.31	8.9 (4.3–22.9)	22	1.00 (0.97–1.04)	0.93
Positive	2.2 (0.9–4.0)	160			11.3 (5.1–20.1)	157		
								
*progesteron receptor †*								
Negative	2.0 (0.2–3.5)	32	1.13 (0.95–1.35)	0.29	12.3 (4.9–23.5)	32	0.98 (0.95–1.00)	0.30
Positive	2.2 (0.9–4.0)	148			10.9 (4.7–19.4)	146		
								
*Her2 overexpression*								
Negative	2.2 (0.8–3.8)	158	0.97 (0.82–1.14)	0.79	10.9 (4.9–20.2)	156	1.03 (1.00–1.06)	0.39
Positive	1.8 (1.1–4.4)	23			13.7 (3.2–25.7)	23		

*p for Mann-Whitney U test or Kruskal-Wallis test. † Progesteron receptor status is unknown for one patient in both MMP1 and MMP8 analyses.

Note: Her2 status was defined by immunohistochemistry and dual-colour FISH analyses

IQR, interquartile range; OR, odds ratio

**Figure 3:**
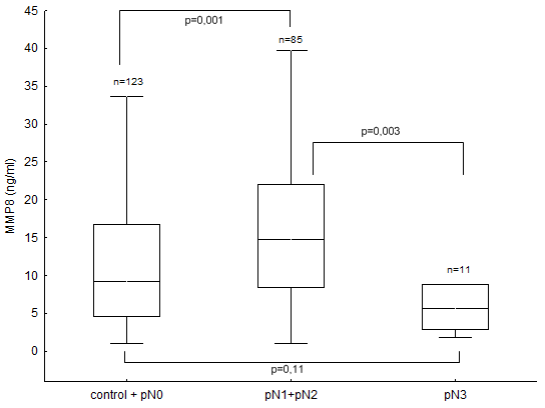
Association of plasma MMP8 levels with the extent of lymph node metastasis and risk of distant metastasis. note: controls + pN0 comprises healthy women and breast cancer patients without detectable lymph node or distant metastasis; pN1+pN2 comprises patients with moderate lymph node metastasis but without distant metastasis; pN3 comprises patients with extensive lymph node metastasis and a high risk of distant metastasis (box-whisker diagrams with median, 1^st ^quartile, 3^rd ^quartile and non-outlier range).

Likewise, the median value of plasma MMP8 levels in the population of non-inflammatory breast cancer patients was almost twice as high as those found in the inflammatory breast cancer group (OR 0.90, p < 0.01; Figure 2b). Further, elevated MMP8 levels weakly correlated with an increasing NPI value (p = 0.04, rs = 0.16; data not shown), and showed a trend with premenopausal status (OR 0.98, p = 0.06). We found an interesting relation between plasma MMP8 levels and lymph node invasion or the risk of distant metastasis. MMP8 levels in controls and lymph node negative patients (pN0) were lower than those in patients with moderate lymph node involvement (pN1, pN2); but higher than those in patients with extensive lymph node metastasis (pN3) and a high risk of distant metastasis, illustrated in Figure 3.

## Discussion

Several studies have reported on the prognostic value of serum and plasma MMP levels in breast cancer. However, this study is, to our knowledge, the first to discuss the prognostic potential of circulating collagenases in breast cancer. The present study clearly demonstrates differences in total MMP-1 and MMP-8 levels in plasma from patients with different clinical and pathological characteristics. Circulating MMP-13 levels did not reach the sensitivity detection limit of the immunoassay and were not further analyzed. MMP-13 plays a key role in collagen remodelling and as such is believed to be involved in local invasion rather than in metastasis formation. It is therefore likely that MMP13 resides in the tumour microenvironment in both early and advanced cancer stages, resulting in low to very low levels entering the circulation.

We found lower plasma MMP1 levels in breast cancer patients than in healthy controls. ROC analysis revealed a AUC of 0.67 with a sensitivity of 80% and specificity of 24% at a cut-off value of 4.24 ng/ml plasma MMP1. This high sensitivity rate suggests that plasma MMP1 may be a good candidate biomarker for breast cancer. Unfortunately, the low specificity of 24% hampers the clinical use of circulating MMP1 due to significant overtreatment of healthy individuals. Nevertheless we cannot exclude that plasma MMP1 measurement in combination with assessment of other candidate or established biomarkers may improve breast cancer diagnosis. Furthermore, we observed lower MMP1 levels in plasma from patients with inflammatory breast cancer, a rare but very aggressive disease, and non-inflammatory breast cancer patients with larger tumours. This seems to be in clear contradiction with previous results demonstrating a clear correlation between MMP1 overexpression and poor prognosis. However, it is not clear yet whether MMPs in blood and body fluids exert any physiological function. Altered MMP levels in blood may simply reflect local changes in the extracellular microenvironment. Thus, we hypothesize that the lower MMP1 levels observed in plasma could reflect a concentration of MMP1 in the tumour microenvironment. As such, the reverse correlation of plasma MMP1 with breast cancer risk and prognosis is to be expected. For instance, the reverse correlation of plasma MMP1 expression with tumour growth is then in accordance with the established tumour growth promoting role of MMP1 in the tumour tissue [21].

We found a trend for higher plasma MMP8 levels in premenopausal patients (p = 0.06). Further, we observed a weak positive correlation (rs = 0.16, p = 0.04) of plasma MMP8 with the Nottingham Prognostic Index which is calculated based on the tumour size, differentiation grade and lymph node status. Since plasma MMP8 did not significantly correlate with either of these parameters, the statistical significance of its relation with the NPI most probably results from a type I error. If we assume, as for MMP1, that the higher plasma MMP8 levels reflect a lower expression in the tumour tissue; then the positive relation of plasma MMP8 with premenopausal status, and as such earlier onset, supports the notion of a protective role of MMP8 in cancer [22-25]. The anti-tumorigenic and anti-metastatic role emphasizes the notion that the role of MMP8 in cancer is far more puzzling than originally thought. Traditionally, MMP8 was considered to promote tumour invasion and metastasis in accordance with the general view on MMPs in cancer. In line with this, elevated levels of MMP8 expression have been observed in head and neck squamous cell carcinomas [26]. In ovarian cancer, MMP8 expression has been associated with tumour grade and stage, as well as with poor prognosis [27,28]. The lower MMP8 levels observed in plasma of patients with inflammatory breast cancer, which may reflect higher levels in the tumour, are consistent with a pro-tumorigenic role of MMP8 and suggest that MMP8 may be implicated in the inflammation-dependent mechanisms of this aggressive breast tumour type.

Our most intriguing finding is the association of plasma MMP8 levels with lymph node metastasis and the risk of distant metastasis. Similar MMP8 expression was found in plasma from healthy controls and breast cancer patients without lymph node involvement (pN0). The median MMP8 levels in controls and lymph node negative patients (pN0) were significantly lower than in patients with moderate lymph node involvement (pN1, pN2); but higher than in patients with extensive lymph node metastasis (pN3) and a strong predisposition to distant metastasis. Based on the hypothesis of reverse association of tissue and blood MMP levels, these results support the anti-metastatic role for MMP8 and may even indicate that MMP8 has a greater protective effect against lymph node metastasis as compared with distant metastasis. This hypothesis has been put forward by Montel and colleagues who recently reported that reducing MMP8 levels by the ribozyme knock-down technique dramatically increased lymph node metastasis of breast cancer cells orthotopically implanted in mice but has a smaller effect on metastasis to the lung [24]. In our study, we observed that 1) higher plasma/lower tumour tissue MMP8 levels were more often found in patients with moderate lymph node involvement and that 2) lower plasma/higher tumour tissue MMP8 levels were more common in patients with pN3 breast disease which is strongly associated with systemic disease. Thus, our results support the data from Montel and coworkers in the observation that MMP8 overexpression in the tumour protects against lymph node metastasis but not against distant metastasis. Moreover, our data indicate that MMP8 overexpression may even have an adverse influence on distant metastasis and may predispose cancer cells to spread by the haematogenous route. This complex association of MMP8 with metastasis may explain why we did not find any significant difference in plasma MMP8 levels between healthy controls and the overall breast cancer group. Nevertheless, it seems unlikely that changes in MMP8 expression alone could affect the metastatic behaviour of cancer cells so dramatically. It is more likely that significant changes in MMP8 expression perturb the constant flux of the protease web, an interconnecting web of different protease classes, families and pathways. Overexpression of one protease may significantly perturb the web, forging new web connections that lead to new, unpredictable activities that may promote or inhibit tumour progression and metastasis [29].

## Conclusion

In conclusion, the present study led us to formulate the hypothesis that blood MMP levels reflect local changes in MMP expression in the tumour microenvironment so that inverse associations are to be expected. Our results support previous data, suggesting a tumour-promoting role for MMP1 in breast cancer. Moreover, the present data exemplify the puzzling dual role of MMP8 in breast cancer as both pro- and anti-tumorigenic/metastatic. Interestingly, we found that MMP8 may affect the metastatic behaviour of breast cancer cells and may be implicated in directing organ-specific metastasis. As MMPs have also important functions in normal tissue homeostasis, one of the challenges in combating cancer metastasis by MMP inhibition is to determine which MMPs are clearly anti-targets. Blocking MMP anti-targets might counterbalance the beneficial effects of target inhibition and have a detrimental influence on patient outcome and mortality, thereby contributing to the failure of MPI clinical trials. Therefore, further investigation with larger patient cohorts is clearly needed to better understand the paradoxical role of MMP8 in cancer metastasis.

## Competing interests

The author(s) declare that they have no competing interests.

## Authors' contributions

JD conceived the study, performed basic statistical analysis, interpreted the data and drafted the manuscript. WH acquired all clinical and pathological data, and contributed to the interpretation of data. UV carried out all immunoassays. VVB designed ROC curves and performed multivariate analyses. SVH critically reviewed the statistical analyses. MRC assisted in the coordination of blood sampling and acquisition of clinical data. SY critically revised the manuscript for important intellectual content. RP coordinated the blood sampling and participated in the design of the study. All authors read and approved the final manuscript.

## Pre-publication history

The pre-publication history for this paper can be accessed here:


